# Assisted reproductive technology in Japan: A summary report for 2022 by the Ethics Committee of the Japan Society of Obstetrics and Gynecology

**DOI:** 10.1002/rmb2.12620

**Published:** 2024-12-15

**Authors:** Yukiko Katagiri, Seung Chik Jwa, Akira Kuwahara, Takeshi Iwasa, Masanori Ono, Keiichi Kato, Hiroshi Kishi, Yoshimitsu Kuwabara, Fuminori Taniguchi, Miyuki Harada, Akira Iwase, Norihiro Sugino

**Affiliations:** ^1^ Department of Obstetrics and Gynecology, Faculty of Medicine Toho University Tokyo Japan; ^2^ Department of Obstetrics and Gynecology Jichi Medical University Tochigi Japan; ^3^ Department of Obstetrics and Gynecology, Graduate School of Biomedical Sciences Tokushima University Tokushima Japan; ^4^ Department of Obstetrics and Gynecology Tokyo Medical University Tokyo Japan; ^5^ Kato Ladies Clinic Tokyo Japan; ^6^ Department of Obstetrics and Gynecology The Jikei University School of Medicine Tokyo Japan; ^7^ Department of Obstetrics and Gynecology Nippon Medical School Tokyo Japan; ^8^ Department of Obstetrics and Gynecology Tottori University Faculty of Medicine Tottori Japan; ^9^ Department of Obstetrics and Gynecology, Graduate School of Medicine The University of Tokyo Tokyo Japan; ^10^ Department of Obstetrics and Gynecology Gunma University Graduate School of Medicine Maebashi Japan; ^11^ Yamaguchi University Graduate School of Medicine Ube Japan

**Keywords:** assisted reproductive technologies, fertility rate, in vitro fertilization, intracytoplasmic sperm injections, Japan

## Abstract

**Purpose:**

This descriptive analysis evaluated the 2022 assisted reproductive technology (ART) data collected by the Japan Society of Obstetrics and Gynecology registry.

**Methods and Results:**

In 2022 (cutoff date 30 November 2023), 634 of 635 registered ART facilities participated; 602 implemented ART treatment, with 543 630 registered cycles and 77 206 neonates (9.1% and 10.6% increases from the previous year). For fresh cycles, freeze‐all in vitro fertilization and intracytoplasmic sperm injection cycles increased, resulting in 2183 and 2822 neonates, respectively. In total, 275 296 cycles resulted in oocyte retrieval, with 158 247 (57.5%) freeze‐all cycles. Total single embryo transfer (ET) and singleton pregnancy rates were 82.4% and 97.2%, respectively. The singleton live birth rate was 97.4%. The number of frozen–thawed ET (FET) cycles was 264 412, with 98 348 pregnancies and 72 201 neonates. The single ET rate was 85.3%. The rate of singleton pregnancies was 96.9%; that of singleton live births was 96.9%. Per registered cycle, women had a mean age of 37.6 (standard deviation: 4.8) years; 210 322 cycles (38.7%) were conducted for women aged ≥40 years.

**Conclusions:**

Significant growth in ART cycles and outcomes reflects the impact of recent expanded insurance coverage.

## Graphical abstract

In 2022, 634 of 635 registered ART facilities participated; 602 implemented ART treatment, with 543 630 registered cycles and 77 206 neonates (9.1% and 10.6% increases from the previous year). Significant growth in ART cycles and outcomes reflects the impact of recent expanded insurance coverage.

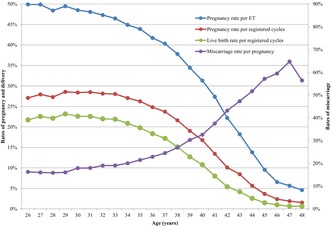

## 1 INTRODUCTION

Fertility rates in Japan have been trending downward over the past four decades,^1^ with rapidly declining birth rates and accelerated aging. By 2020, the total fertility rate in Japan had decreased to 1.33 births per woman,^2^ lower than the previous record of 1.36 in 2019 and significantly down from the 1.44 rate in 2016.^2^ More recent data indicate that the total fertility rate in Japan has continued to decrease yearly to historically low rates of 1.26 and 1.20 births per woman in 2022 and 2023.^3^ The World Bank reported a global fertility rate of 2.4 in 2019, 2.3 in 2020, and 2.3 in 2022, depicting a similar global trend in declining fertility rates.^4^ The underlying causes of this phenomenon are complex, with a range of factors thought to have impacted fertility and birth rates in Japan. These may include tendencies to marry late or not at all,^5^, ^6^ increasing trends in later childbearing that accompany women's empowerment in education and the workforce,^7^ increased burdens of parenting and rising costs of raising children, difficulties women experience in continuing to work,^8^ increases in the rate of irregular employment,^9^, ^10^ and growth of a super‐aged population.^11^, ^12^

The Japanese government has made extensive efforts to reverse these fertility trends, among which perhaps the most impactful measures might be the doubling of government spending on child‐related programs and coverage of assisted reproductive technology (ART) and male infertility treatments by public insurance since April 2022.^13^, ^14^ Given the increasing trend toward later childbearing, Japan's ART field has seen significant advancements and changes over the years, reflecting evolving societal attitudes and advancements in medical technology.^14^, ^15^ Indeed, Japan is a leading country in the use of ART.^16^ In 2021, 498 140 cycles of ART were performed in Japan, which led to 69 797 live births, representing increases of 10.7% and 15.5%, respectively, from the numbers reported in 2020.^17^

The Ethics Committee of the Japan Society of Obstetrics and Gynecology (JSOG) has been monitoring and reporting developments in ART since 1986. In 2007, it implemented an online ART registration system. The committee publishes an annual report that provides a comprehensive overview of ART practices, trends, and ethical considerations in Japan. This examination of data from registered ART facilities may be helpful in updating policymakers, health care providers, and the public about the evolving landscape of reproductive medicine. The following report will examine the detailed findings and implications of the 2022 ART data collected by the JSOG and compare the present results with those from previous years.

## 2 MATERIALS AND METHODS

### 2.1 Data source and data collection

The JSOG registry collects data from registered ART facilities across Japan. It collects demographic and background characteristics of patients, clinical information such as infertility diagnosis, treatment information, and pregnancy and obstetric outcomes following treatment as ART‐cycle‐specific data.^18^ The present descriptive analysis investigated registered cycle characteristics and treatment outcomes using data from the Japanese ART registry in 2022 with a cutoff date of 30 November 2023.

### 2.2 Variables of interest

Data for the following variables by fertilization method (in vitro fertilization [IVF], intracytoplasmic sperm injection [ICSI], and frozen–thawed embryo transfer [FET]) were collected, analyzed, and compared with data from previous years: number of registered cycles, oocyte retrievals, embryo transfer (ET) cycles, freeze‐all‐embryo/oocyte cycles, and numbers of pregnancies and neonates. Characteristics of registered cycles and pregnancy outcomes were described for fresh and FET cycles. Fresh cycle data were stratified by fertilization method (i.e., IVF and ICSI).

### 2.3 Outcomes

The list and definitions of the treatment outcomes analyzed and compared were as follows: pregnancy (confirmation of a gestational sac in utero), miscarriage (spontaneous or unplanned loss of a fetus from the uterus before 22 weeks of gestation), live birth (delivery of at least one live neonate after 22 weeks of gestation), and multiple pregnancy rates.

The pregnancy outcomes analyzed and compared were ectopic pregnancy, heterotopic pregnancy, artificially induced abortion, stillbirth, and fetal reduction. The following outcomes were also analyzed by patient age: pregnancy, live birth, miscarriage, and multiple pregnancy rates. Treatment outcomes for FET cycles using frozen–thawed oocytes were also analyzed.

### 2.4 Statistical analysis

All analyses were conducted using the STATA MP statistical package, version 18.5 (Stata, College Station). Statistical testing was not conducted as this study focuses on descriptive analysis.

## 3 RESULTS

In 2022, of the 635 registered ART facilities, 634 participated in the JSOG registry and, of these, 602 actually implemented ART treatment.

Table 1 summarizes the main trends in the numbers of registered cycles, egg retrievals, pregnancy, and neonate births categorized by IVF, ICSI, and FET cycles in Japan (2007–2022). In 2022, 543 630 cycles were registered for IVF, ICSI, and FET, and a total of 77 206 neonates were recorded in Japan, representing 9.1% and 10.6% increases from the previous year. Of note, the number of IVF cycles registered increased by 3.4%, and ICSI cycles increased by 10.3% from the numbers reported in 2021.

**TABLE 1:** Trends in numbers of registered cycles, oocyte retrieval, pregnancy, and neonates based on IVF, ICSI, and frozen–thawed embryo transfer cycles in Japan, 2007–2022.

Year	IVF^a^						ICSI^b^						FET cycle^c^			
	No. of registered cycles	No. of egg retrievals	No. of freeze‐all cycles	No. of ET cycles	No. of cycles with pregnancy	No. of neonates	No. of registered cycles	No. of egg retrievals	No. of freeze‐all cycles	No. of ET cycles	No. of cycles with pregnancy	No. of neonates	No. of registered cycles	No. of ET cycles	No. of cycles with pregnancy	No. of neonates
2007	53 873	52 165	7626	28 228	7416	5144	61 813	60 294	11 541	34 032	7784	5194	45 478	43 589	13 965	9257
2008	59 148	57 217	10 139	29 124	6897	4664	71 350	69 864	15 390	34 425	7017	4615	60 115	57 846	18 597	12 425
2009	63 083	60 754	11 800	28 559	6891	5046	76 790	75 340	19 046	35 167	7330	5180	73 927	71 367	23 216	16 454
2010	67 714	64 966	13 843	27 905	6556	4657	90 677	88 822	24 379	37 172	7699	5277	83 770	81 300	27 382	19 011
2011	71 422	68 651	16 202	27 284	6341	4546	102 473	100 518	30 773	38 098	7601	5415	95 764	92 782	31 721	22 465
2012	82 108	79 434	20 627	29 693	6703	4740	125 229	122 962	41 943	40 829	7947	5498	119 089	116 176	39 106	27 715
2013	89 950	87 104	25 085	30 164	6817	4776	134 871	134 871	49 316	41 150	8027	5630	141 335	138 249	45 392	32 148
2014	92 269	89 397	27 624	30 414	6970	5025	144 247	141 888	55 851	41 437	8122	5702	157 229	153 977	51 458	36 595
2015	93 614	91 079	30 498	28 858	6478	4629	155 797	153 639	63 660	41 396	8169	5761	174 740	171 495	56 888	40 611
2016	94 566	92 185	34 188	26 182	5903	4266	161 262	159 214	70 387	38 315	7324	5166	191 962	188 338	62 749	44 678
2017	91 516	89 447	36 441	22 423	5182	3731	157 709	155 758	74 200	33 297	6757	4826	198 985	195 559	67 255	48 060
2018	92 552	90 376	38 882	20 894	4755	3402	158 859	157 026	79 496	29 569	5886	4194	203 482	200 050	69 395	49 383
2019	88 074	86 334	40 561	17 345	4002	2974	154 824	153 014	83 129	24 490	4789	3433	215 203	211 758	74 911	54 188
2020	82 883	81 286	42 530	13 362	3094	2282	151 732	150 082	87 697	19 061	3626	2596	215 285	211 914	76 196	55 503
2021	88 362	86 901	42 016	13 219	3115	2268	170 350	168 659	86 992	19 740	3875	2850	239 428	236 211	87 174	64 679
2022	91 402	89 807	49 433	12 211	3007	2183	187 816	185 489	108 814	19 299	3878	2822	264 412	260 101	98 348	72 201

Abbreviations: ET, embryo transfer; FET, frozen–thawed embryo transfer; GIFT, gamete intrafallopian transfer; ICSI, intracytoplasmic sperm injection; IVF, in vitro fertilization.

^a^ Including GIFT and other.

^b^ Including split‐ICSI cycles.

^c^ Including cycles using frozen–thawed oocyte.

In contrast with 2021, freeze‐all IVF and ICSI increased by 17.7% and 25.1%, respectively. The number of neonates born by IVF‐ET cycles was 2183 and 2822 by ICSI, representing slight decreases (3.7% and 1.0%) from the previous year. The continuously increasing trend seen for FET cycles since 2007 was maintained in 2022, with a 10.4% increase. The number of FET cycles was 264 412, with 98 348 pregnancies and 72 201 neonates.

**FIGURE 1:**
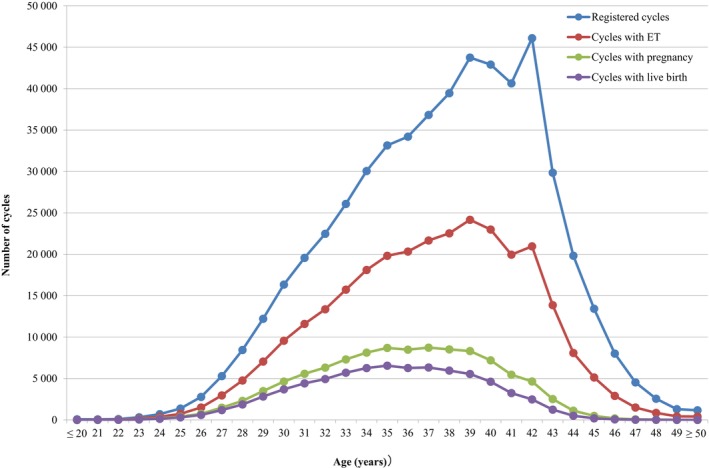
Distribution of maternal age from all registered cycles, cycles for ET, cycles leading to pregnancy, and cycles leading to live births in 2022. Adapted from the Japan Society of Obstetrics and Gynecology ART Databook 2022 (https://www.jsog.or.jp/activity/art/2022_JSOG‐ART.pdf). ET, embryo transfer.

Figure 1 shows the age distributions for all registered cycles and different subgroups of cycles for ET, pregnancy, and live births in 2022. The mean patient age for registered cycles was 37.6 years (standard deviation [SD] ± 4.8); the mean age for pregnancy and live birth cycles was 35.7 years (SD ± 4.3) and 35.2 years (SD ± 4.2), respectively. In 2022, 38.7% of ART cycles (210 322 cycles) registered were undertaken for women aged 40 years or over. Of note, there was a peak in registered cycles (46095) among patients aged 42 years.

### 3.1 Treatment and pregnancy outcomes

The detailed characteristics and treatment outcomes of registered fresh cycles are shown in Table 2. In 2022, 85 124 IVF cycles, 34 581 split‐ICSI cycles, 150 958 ICSI cycles using ejaculated spermatozoa, 2277 ICSI cycles using testicular sperm extraction (TESE), 2628 cycles for oocyte freezing, and 3650 other cycles were registered. In total, 275 296 cycles resulted in oocyte retrieval, of which 158 247 (57.5%) were freeze‐all cycles. The pregnancy rate was 24.6% per ET cycle of IVF, and 19.2% for ICSI using ejaculated spermatozoa. The total single ET rate was 82.4%, and the pregnancy rate following a single ET cycle was 22.6%. Live birth rates per ET were 17.4% for IVF, 19.0% for split‐ICSI, 13.5% for ICSI using ejaculated spermatozoa, and 8.6% for ICSI with TESE. There were 6556 singleton pregnancies and 4758 singleton live births. In 2022, 2628 cycles for oocyte freezing were registered, and 2608 oocyte retrievals were conducted. Of these, 2402 cycles led to successfully frozen oocytes. The singleton pregnancy rate was 97.2%, and the singleton live birth rate was 97.4%.

**TABLE 2:** Characteristics and treatment outcomes of registered fresh cycles in assisted reproductive technology in Japan, 2022.

Variables	IVF	Split‐ICSI	ICSI		Frozen oocyte	Other^a^	Total
			Ejaculated sperm	TESE			
No. of registered cycles	85 124	34 581	150 958	2277	2628	3650	279 218
No. of egg retrievals (0 or more)	83 586	34 293	148 923	2273	2608	3613	275 296
No. of fresh ET cycles (1 or more)	11 951	2907	16 088	304	0	260	31 510
No. of freeze‐all cycles	45 068	27 010	80 436	1368	2402	1963	158 247
No. of cycles with pregnancy	2942	752	3084	42	0	65	6885
Pregnancy rate per ET	24.6%	25.9%	19.2%	13.8%		25.0%	21.9%
Pregnancy rate per egg retrieval	3.5%	2.2%	2.1%	1.9%		1.8%	2.5%
Pregnancy rate per egg retrieval excluding freeze‐all cycles	4.5%	3.6%	2.7%	2.6%		2.0%	3.3%
SET cycles	10 321	2529	12 721	186		220	25 977
Pregnancy following SET cycles	2586	686	2515	32		61	5880
Rate of SET cycles	86.4%	87.0%	79.1%	61.2%		84.6%	82.4%
Pregnancy rate following SET cycles	25.1%	27.1%	19.8%	17.2%		27.7%	22.6%
Miscarriages	709	158	785	14		12	1678
Miscarriage rate per pregnancy	24.1%	21.0%	25.5%	33.3%		18.5%	24.4%
Singleton pregnancies^b^	2801	720	2931	41		63	6556
Multiple pregnancies^b^	74	20	90	0		2	186
Twin pregnancies	73	20	88	0		0	183
Triplet pregnancies	1	0	2	0		0	3
Quadruplet pregnancies	0	0	0	0		0	0
Multiple pregnancy rate	2.6%	2.7%	3.0%	0.0%		3.1%	2.8%
Live births	2082	553	2172	26		50	4883
Live birth rate per ET	17.4%	19.0%	13.5%	8.6%		19.2%	15.5%
Total no. of neonates	2133	568	2228	26		50	5005
Singleton live births	2031	538	2113	26		50	4758
Twin live births	51	15	56	0		0	122
Triplet live births	0	0	1	0		0	1
Quadruplet live births	0	0	0	0		0	0
Ectopic pregnancies	39	4	39	1		1	84
Heterotopic pregnancies	1	0	0	0		0	1
Artificial abortions	11	4	19	1		1	36
Still births	12	1	9	0		0	22
Fetal reductions	0	0	1	0		0	1
Cycles with unknown pregnancy outcomes	57	27	47	0		1	132

Abbreviations: ET, embryo transfer; ICSI, intracytoplasmic sperm injection; IVF, in vitro fertilization; SET, single embryo transfer; TESE, testicular sperm extraction; ZIFT, zygote intrafallopian transfer.

^a^ Others include ZIFT.

^b^ Singleton, twin, triplet, and quadruplet pregnancies were defined on the basis of the number of gestational sacs in utero.

Table 3 summarizes the characteristics and treatment outcomes of FET cycles. In 2022, a total of 264 015 cycles were registered. Of these, 262 146 were registered as FET cycles. Of the latter, 258 217 FETs were conducted. With a pregnancy rate of 37.8%, FET cycles resulted in 97 664 pregnancies. FET cycles resulted in 24 969 miscarriages. The miscarriage rate per pregnancy was 25.6%, and the live birth rate per FET increased slightly to 27.0% from 26.6% observed in 2021. The single ET rate was 85.3%, somewhat higher than in 2021 (84.9%), resulting in a slightly increased pregnancy rate of 38.8% from 38.1% in 2021. The rate of singleton pregnancies was 96.9%, and singleton live births was 96.9%.

**TABLE 3:** Characteristics and treatment outcomes of frozen cycles in assisted reproductive technology in Japan, 2022.

Variables	FET	Other^a^	Total
No. of registered cycles	262 146	1869	264 015
No. of FET	258 217	1688	259 905
No. of cycles of pregnancy	97 664	643	98 307
Pregnancy rate per FET	37.8%	38.1%	37.8%
SET cycles	220 292	1386	221 678
Pregnancy following SET cycles	85 432	538	85 970
Rate of SET cycles	85.3%	82.1%	85.3%
Pregnancy rate following SET cycles	38.8%	38.8%	38.8%
Miscarriages	24 969	181	25 150
Miscarriage rate per pregnancy	25.6%	28.2%	25.6%
Singleton pregnancies^b^	93 406	617	94 023
Multiple pregnancies^b^	3000	16	3016
Twin pregnancies	2939	16	2955
Triplet pregnancies	54	0	54
Quadruplet pregnancies	6	0	6
Quintuplet pregnancies	1	0	1
Multiple pregnancy rate	3.1%	2.5%	3.1%
Live births	69 834	435	70 269
Live birth rate per FET	27.0%	25.8%	27.0%
Total no. of neonates	71 733	446	72 179
Singleton live births	67 646	424	68 070
Twin live births	2018	11	2029
Triplet live births	17	0	17
Quadruplet live births	0	0	0
Ectopic pregnancies	476	1	477
Heterotopic pregnancies	23	0	23
Artificial abortions	436	4	440
Stillbirths	239	5	244
Fetal reductions	18	0	18
Cycles with unknown pregnancy outcomes	1430	8	1438

Abbreviations: FET, frozen–thawed embryo transfer; SET, single embryo transfer.

^a^ Including cycles using frozen–thawed oocytes.

^b^ Singleton, twin, triplet, and quadruplet pregnancies were defined on the basis of the number of gestational sacs in utero.

### 3.2 Outcomes by patient age

Table 4 shows the treatment outcomes of registered cycles by patient age in Japan in 2022. The pregnancy rate per ET exceeded 40% for women aged between 21 and 37 years. Gradual decreases in pregnancy rates per ET were observed with increasing maternal age, starting at age 26 years. Rates fell below 30% for women aged >41 years, below 20% among women aged >43 years, below 10% for women aged >45 years, and below 5% for women aged >48 years. The miscarriage rates tended to be below 20% for all women aged between 22 and 34 years and increased gradually with increasing maternal age. Women in their early forties had miscarriage rates generally between 33% and 52%, while women in their mid‐forties had miscarriage rates over 57%. The live birth rate per registered cycle was the highest for women aged 29 years (23.2%). Rates declined sharply to below 15.0% at 39 years of age and below 10.0% among women >41 years of age.

**TABLE 4:** Treatment outcomes of registered cycles based on patient age in Japan, 2022.

Age (years)	No. of registered cycles	No. of ET cycles	No. of cycles with pregnancy	Multiple pregnancies	Miscarriage	Cycles with live birth	Pregnancy rate/registered ET (%)	Pregnancy rate/registered cycles (%)	Live birth rate/registered cycles	Miscarriage rate (%)	Multiple pregnancy rate (%)^a^
≤20	80	8	4	0	1	3	50.0	5.0	3.8	25.0	0.0
21	73	32	13	2	2	9	40.6	17.8	12.3	15.4	8.3
22	106	46	25	1	7	18	54.4	23.6	17.0	28.0	4.0
23	321	155	72	3	10	60	46.5	22.4	18.7	13.9	4.2
24	704	379	182	6	37	141	48.0	25.9	20.0	20.3	1.7
25	1375	730	386	24	50	317	52.9	28.1	23.1	13.0	3.5
26	2777	1507	752	20	122	603	49.9	27.1	21.7	16.2	2.0
27	5290	2961	1477	72	236	1193	49.9	27.9	22.6	16.0	3.4
28	8452	4764	2306	92	365	1869	48.4	27.3	22.1	15.8	2.7
29	12 217	7054	3489	148	559	2831	49.5	28.6	23.2	16.0	3.1
30	16 342	9563	4639	196	830	3692	48.5	28.4	22.6	17.9	3.3
31	19 571	11 596	5574	215	1000	4415	48.1	28.5	22.6	17.9	2.7
32	22 481	13 366	6323	236	1201	4939	47.3	28.1	22.0	19.0	2.5
33	26 083	15 732	7312	312	1391	5704	46.5	28.0	21.9	19.0	3.0
34	30 060	18 109	8132	319	1628	6268	44.9	27.1	20.9	20.0	2.9
35	33 153	19 818	8702	394	1867	6558	43.9	26.3	19.8	21.5	3.0
36	34 198	20 337	8486	392	1940	6271	41.7	24.8	18.3	22.9	3.3
37	36 825	21 664	8734	389	2138	6335	40.3	23.7	17.2	24.5	3.2
38	39 450	22 535	8522	416	2290	5960	37.8	21.6	15.1	26.9	3.6
39	43 750	24 167	8320	377	2517	5550	34.4	19.0	12.7	30.3	3.4
40	42 903	22 990	7199	337	2344	4616	31.3	16.8	10.8	32.6	3.3
41	40 639	19 954	5460	231	2047	3249	27.4	13.4	8.0	37.5	3.3
42	46 095	20 960	4651	219	2007	2484	22.2	10.1	5.4	43.2	3.2
43	29 849	13 859	2524	98	1194	1246	18.2	8.5	4.2	47.3	2.6
44	19 824	8085	1116	45	577	508	13.8	5.6	2.6	51.7	2.5
45	13 425	5131	490	13	280	197	9.6	3.6	1.5	57.1	1.7
46	8019	2908	190	7	113	77	6.5	2.4	1.0	59.5	1.6
47	4542	1506	85	3	55	29	5.6	1.9	0.6	64.7	0.0
48	2561	851	39	1	22	16	4.6	1.5	0.6	56.4	0.0
49	1302	432	17	2	8	8	3.9	1.3	0.6	47.1	11.8
≥50	1163	412	12	1	6	6	2.9	1.0	0.5	50.0	0.0
Total	543 630	291 611	105 233	4571	26 844	75 172	36.1	19.4	13.8	25.5	3.1

Abbreviation: ET, embryo transfer.

^a^ Multiple pregnancies were defined on the basis of the number of gestational sacs in utero.

**FIGURE 2:**
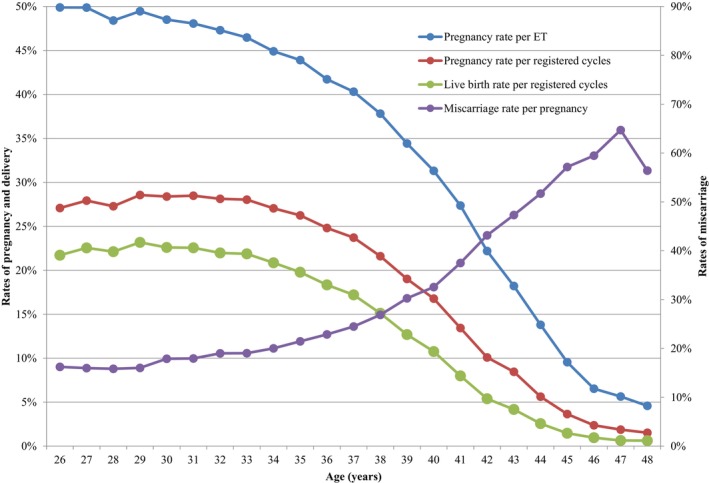
Pregnancy, live birth, and miscarriage rates according to patient age in all registered cycles 2022. Adapted from the Japan Society of Obstetrics and Gynecology ART Databook 2022 (https://www.jsog.or.jp/activity/art/2022_JSOG‐ART.pdf). ET, embryo transfer.

Figure 2 shows the rates of pregnancy, live birth, and miscarriage by patient age in all registered cycles in 2022. Of note, the pregnancy rate per ET was around 50% at ages 26 and 27 and generally above 45% between ages 28 and 34 years. There was then a progressive decline from that point, which became even more marked beyond the age of 40 years, similar to that reported in the previous year. Similar trends were observed for pregnancy and live birth rates (below 30% and 25%, respectively), with progressive declines starting as early as 35 years of age. Conversely, miscarriage rates gradually increased from the early thirties up to 38 years of age and increased rapidly thereafter until the late forties.

### 3.3 Treatment outcomes for FET cycles using frozen–thawed oocytes

Table 5 shows the primary treatment outcomes of embryo transfers using frozen–thawed oocytes in Japan in 2022. In 2022, 397 cycles using frozen–thawed oocytes were registered in Japan, of which 196 FETs were actually implemented. Forty‐one pregnancies were achieved, with a pregnancy rate per FET of 20.9% and a live birth rate of 10.2%. The miscarriage rate per pregnancy was 39.0%.

**TABLE 5:** Treatment outcomes of embryo transfers using frozen–thawed oocyte in assisted reproductive technology in Japan, 2022.

Variables	Embryo transfers using frozen–thawed oocytes
No. of registered cycles	397
No. of ET	196
No. of cycles with pregnancy	41
Pregnancy rate per ET	20.9%
SET cycles	120
Pregnancy following SET cycles	29
Rate of SET cycles	61.2%
Pregnancy rate following SET cycles	24.2%
Miscarriages	16
Miscarriage rate per pregnancy	39.0%
Singleton pregnancies^a^	36
Multiple pregnancies^a^	1
Twin pregnancies	1
Triplet pregnancies	0
Quadruplet pregnancies	0
Multiple pregnancy rate	2.7%
Live births	20
Live birth rate per ET	10.2%
Total number of neonates	22
Singleton live births	18
Twin live births	2
Triplet live births	0
Quadruplet live births	0
Ectopic pregnancies	0
Intrauterine pregnancies coexisting with ectopic pregnancy	0
Artificial abortions	1
Still births	1
Fetal reductions	0
Cycles with unknown pregnancy outcomes	2

Abbreviations: ET, embryo transfer; SET, single embryo transfer.

^a^ Singleton, twin, triplet, and quadruplet pregnancies were defined on the basis of the number of gestational sacs in utero.

## 4 DISCUSSION

We described the characteristics and outcomes of ART cycles registered in the Japanese ART registry system during 2022 and compared the present results with those from 2021^17^ and previous years.^19^, ^20^, ^21^, ^22^ The main findings of the Japanese ART registry in 2022 were as follows: in 2022, 543 630 cycles were registered; 105 233 pregnancies and a total of 77 206 neonate births were recorded by the JSOG in Japan.

In 2022, there were significant increases in ART cycles. IVF cycles increased by 3.4%, and ICSI cycles increased by 10.3%. Freeze‐all cycles accounted for 57.5% of cycles with oocyte retrieval, resulting in a 3.7% decrease in neonates born from IVF‐ET cycles and a 1.0% decrease in those born from ICSI cycles. FET cycles also increased by 10.4%. A total of 210 322 cycles (38.7%) were for cycles in women aged 40 years or over. The total single ET and singleton pregnancy rates for fresh cycles were 82.4% and 97.2%, respectively, and the singleton live birth rate was 97.4%. For frozen cycles, the single ET rate was 85.3%. The rates of singleton pregnancies and singleton live births were both 96.9%.

This report also reflects the impact of the first year since the expansion of insurance coverage for ART (April 2022). This expansion is perhaps the most impactful influence on the increase in the number of ART treatments in Japan, with an increased number of cycles and live births in 2022 (543 630 and 77 206, respectively) compared with 2021 (498 140 and 69 797, respectively).^17^ This coverage marks a significant improvement in access to fertility treatments in Japan. It not only alleviates the financial burden on patients but also represents a crucial step toward equity in reproductive health care. For low‐income couples who aspire to become parents, the cost of ART can be prohibitively high, often leading to emotional distress and limiting their options. With insurance coverage, these couples can pursue treatments without the constant worry of overwhelming expenses, thereby fostering a more supportive environment for family planning. In addition, young couples, who may be navigating the challenges of establishing their careers and finances, also stand to benefit significantly. By reducing the out‐of‐pocket costs associated with ART, insurance coverage enables them to make informed decisions about starting a family without the immediate pressure of financial constraints.

Additionally, the implementation of the “High‐cost Medical Expense Benefit” is a noteworthy aspect of this initiative. If the copayment, calculated on the basis of certain standards, exceeds the maximum, the excess amount will be paid as High‐cost Medical Care Benefits. This program provides further financial support to individuals who face very high medical expenses, ensuring that those requiring extensive ART services are not unduly burdened.^23^ By minimizing the financial risks associated with fertility treatments, this benefit can enhance treatment adherence and, ultimately, improve reproductive outcomes.

Some patients may face greater financial strain, even under the new insurance coverage system. Several local governments have started offering subsidies for advanced ART treatments not covered by public insurance. Such treatments are combined with ART procedures and are usually paid for entirely by the patient. The effect of those additional subsidies—especially for boosting the fertility rate—are, as yet, unknown. Despite being the most accessible region for ART treatments, Tokyo has the lowest fertility rate.^24^ This suggests that simply reducing the financial burden of ART may not be enough to improve fertility trends.

The current system is well organized, but concerns have been raised about developing new ART treatments. Individual clinics usually innovate and develop new ART treatments, but insurance coverage seems to focus on standardized procedures. This could be, in part, because standardized treatments have established success rates and are easier to regulate and cover under insurance policies. As new treatments emerge, integrating them into the existing system, which currently leans toward standard ART, may pose certain challenges.

Another important factor that may limit families from receiving the ART insurance coverage benefit is that the couple's relationship is also scrutinized.^25^ In Japan, there is no specific legislation governing the use of third‐party gametes or embryos for ART. JSOG provides guidelines, but these are not legally binding.^26^, ^27^ Thus, ongoing discussion is needed regarding the creation of more comprehensive regulations.^28^

In 2022, out of 2628 oocyte freezing cycles, 2402 resulted in successfully frozen oocytes, while in 2021, out of 1103 cycles, 830 resulted in the successful freezing of oocytes. This represents success rates of approximately 91.4% in 2022 and approximately 75.2% in 2021, indicating a considerable increase in the success rate of oocyte freezing from 2021 to 2022.^17^

Several factors could contribute to this improvement. Fertility preservation in Japan, especially for medical reasons such as cancer, has become more popular. The Japanese government has established subsidy systems to support this. Patients can apply for subsidies from both local and central governments to help cover the costs of fertility preservation and subsequent ART.^29^, ^30^ Advances in cryopreservation techniques, such as vitrification, have improved oocyte survival rates during freezing and thawing,^31^, ^32^ with live birth rates varying based on the age at which oocytes were frozen.^31^ The higher number of oocyte freezing in 2022 compared with 2021 underscores the positive impact of both technological advancements and diffusion of fertility preservation using ART in Japan.

The pregnancy rate per FET cycle has shown a secular trend, with a slight increase from 36.9% in 2021 to 37.8% in 2022. This trend is an interesting finding and might be influenced by the introduction of preimplantation genetic testing for aneuploidy (PGT‐A) in Japan, following a clinical trial conducted by the JSOG.^33^ PGT‐A helps select chromosomally normal embryos, potentially improving implantation and pregnancy rates per embryo transfer.^34^ Because of this technique, the single ET rate might increase for FET. In the future, it may be beneficial to assess pregnancy rates separately by PGT‐A status in FET cycles.

This study has some strengths and limitations that have been previously reported.^17^

The main strength is that registered ART facilities nationwide must provide annual reports, leading to high reporting compliance. Furthermore, the standardization of procedures and definitions for cycle‐specific information across registered ART facilities has reduced reporting bias. A major limitation is that some data for which collection is not standardized, such as background information, may be more likely incomplete or missing. Furthermore, the registration procedure is somewhat cumbersome in that participating ART facilities are assumed to register cycle‐specific information manually one‐by‐one. Therefore, it is possible that burdens relating to data input are very high and that errors might occur. To address this, the JSOG has launched a subcommittee to debate an effective registration system from 2024, and aims to introduce a batch registration system in the near future.

The 2022 ART data analysis from the Japanese ART registry administered by the JSOG highlights significant growth in ART cycles and outcomes, reflecting the impact of the recent expansion of insurance coverage. Despite the increase in ART cycles, success rates and outcomes vary by age, emphasizing the need for continued advancements and monitoring regarding ART treatments. The data underscore the importance of age in ART outcomes, with higher pregnancy and live birth rates among younger age groups. The expansion of insurance coverage and local government subsidies have contributed to a notable increase in ART use in Japan. However, financial strain and regional disparities in fertility rates suggest that further measures are needed to address underlying challenges and improve overall fertility trends. This annual analysis is essential to comprehending the changing trends and patterns in ART, especially given the continuously declining fertility rate, growing elderly population, and decreasing population growth worldwide, particularly in Japan. As Japan continues to lead ART, integrating new treatments into the standardized insurance‐covered procedures will be crucial. Addressing the financial and logistical barriers faced by patients, especially in regions with lower fertility rates, will be essential for sustaining and enhancing the success of ART programs.

## CONFLICT OF INTEREST STATEMENT

The authors have no conflict of interest to disclose about the present work. “Seung Chik, Jwa”, “Akira, Iwase”, “Takeshi, Iwasa”, are an Editorial Board member of Reproductive Medicine and Biology and a coauthor of this article. To minimize bias, they were excluded from all editorial decision‐making related to the acceptance of this article for publication.

## HUMAN RIGHTS STATEMENTS AND INFORMED CONSENT

All procedures were performed according to the ethical standards of the relevant committees on human experimentation (institutional and national), as well as the Helsinki Declaration of 1964 and its later amendments.

## ANIMAL RIGHTS

This report contains no studies performed by any authors that included animals.
